# In-situ microscopy and digital image correlation to study the mechanical characteristics of polymer-based materials

**DOI:** 10.1007/s43939-025-00208-8

**Published:** 2025-02-18

**Authors:** Seyedtaghi Mousavi, John G. Hardy

**Affiliations:** 1 Department of Biochemistry, Payame Noor University, P. O. Box 19395-3697, Tehran, Iran; 2 Department of Chemistry, Lancaster University, Lancaster, Lancashire LA1 4YB UK; 3 Materials Science Lancaster, Lancaster University, Lancaster, Lancashire LA1 4YB UK

**Keywords:** Composites, Digital image correlation, In-situ optical microscopy, In-situ non-optical microscopy, Materials characterization

## Abstract

In-situ microscopic methods can help researchers to analyse microstructural changes of materials structures under different conditions (e.g., temperature and pressure) at various length scales. Digital Image Correlation (DIC) combines image registration and tracking to enable accurate measurements of changes in materials in 2D and 3D. This review focuses on combining microscopy and DIC to study the properties of materials (including natural/synthetic biomaterials, biological samples and their composites) in academic, public and industry settings, including exciting examples of bioimaging.

## Article highlights

- In-situ microscopic methods facilitate understanding of microstructural changes of materials.
- The DIC technique can be used with optical and non-optical microscopic methods.
- The DIC technique is applicable for a broad range of length scales and time scales.
- The techniques are applicable for a variety of soft and hard materials and their composites, including natural/biological materials.

## Introduction

Polymer composites are ubiquitous in our everyday lives because of their functional/mechanical properties [1], the mechanical properties of such materials are underpinned by the nanoscale/microscale features constituting the structures [2], and there are some excellent reviews on this topic [3–7]. Traditional mechanical testing methods obtain information about the macroscopic physical properties of polymers and their composites, and it is important to note that that can miss information about the contribution of the nanoscale/microscale structures present in these materials [8], and there is significant interest in applying such methods to analyzing biological samples (particularly to assess cell mechanics) [9]. Correlation of multi-scale structures and macroscopic properties is an area of current analytical research [10, 11]; a variety of different laboratory and computational techniques can be employed to understand the behavior of polymers and their composites [12–15]. Methods for mechanical characterization (e.g., compressive, tensile, rheology, etc.) at various length scales coupled with DIC have been employed to analyze polymer-based materials (including composites) undergoing large deformations [16–18]; e.g., polylactide-based materials [19], the thermoset elastomer polyurea [20], shape-memory polymers [21], 3D printed polymeric metamaterials [22], all cellulose composites [23], nitrile rubber composites [24], fiber reinforced polypropylene composites [25], glass fiber reinforced thermoplastics [26], carbon black-silicone composites [27], polymer fiber reinforced concrete [28].

**Fig. 1:**
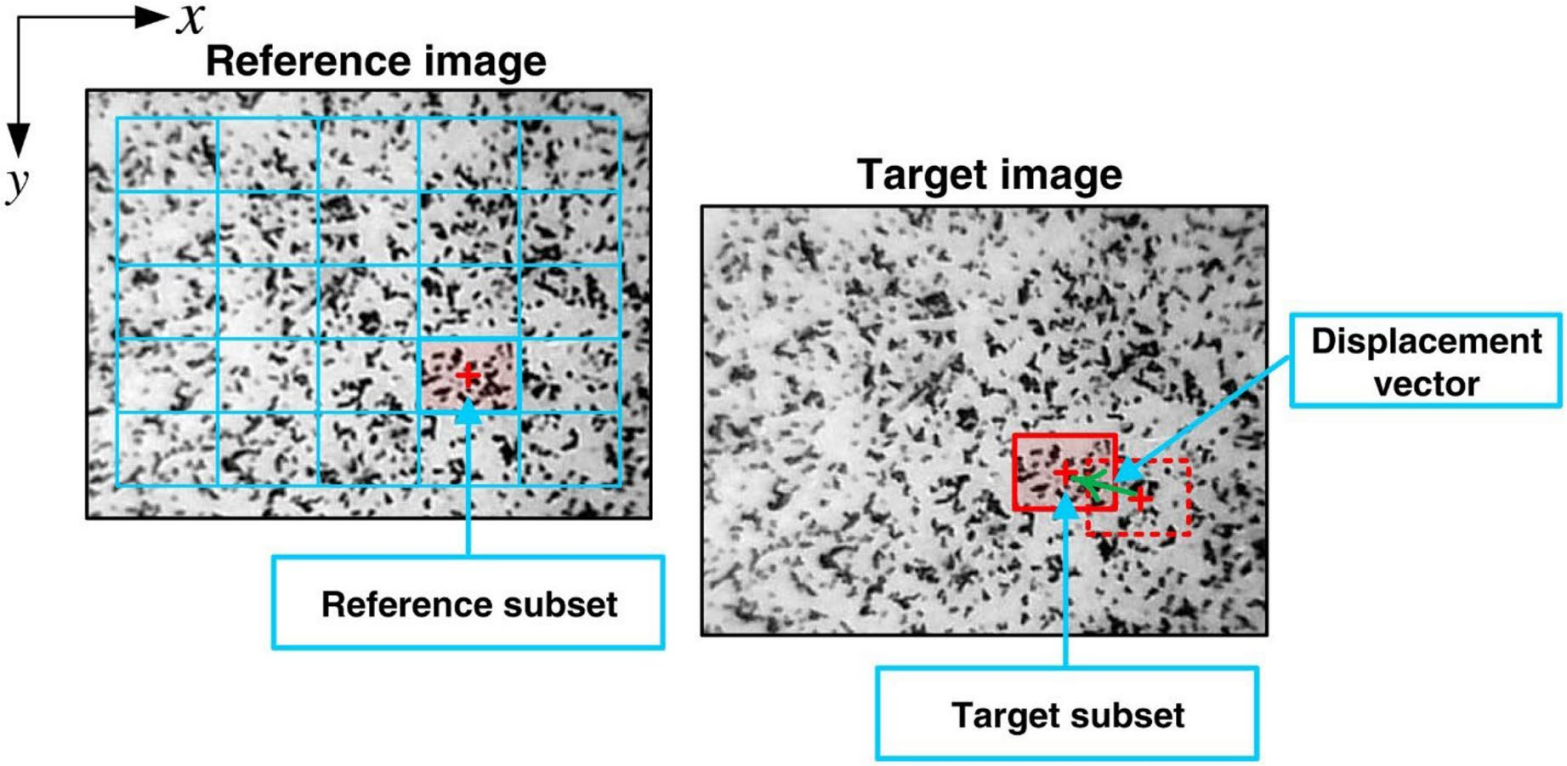
Schematic illustration of the undeformed subset and the corresponding deformed subset in 2D-DIC. Reproduced from [29] with permission from the publisher (John Wiley and Sons)

The DIC technique (Fig. 1) [29] is one of the most frequently exploited methods used with optical and non-optical microscopic methods (e.g., atomic force microscopy [AFM], scanning electron microscopy [SEM], etc.) for measurements from the nm to cm scale, potentially employing both 2D DIC (with a single camera) and 3D DIC or stereo DIC (with 2 synchronized cameras) to achieve high-spatial-resolution imaging [16, 17, 30]. The fundamental DIC procedure involves application of a speckle pattern to a surface, capturing a series of digital images during mechanical testing, followed by DIC analysis to determine displacements/strains on the surface [30–32].

A significant challenge in all DIC applications (including local DIC and global DIC [GDIC]) involves creating optimized DIC patterns for specimens via preparation of speckle patterns on test sample surfaces [16, 17, 30–34]; the macroscale DIC patterns are mostly random grey scale patterns that are deposited via a variety of techniques [30, 34, 35]; for nanoscale patterning drop casting or spraying droplets of nanoparticle-loaded solvents are popular, however, the homogeneity of particle density is a challenge because they tend to cluster [35–39]. In correlative imaging samples are studied through two or more techniques with images located in the same field of view yielding greater insights than any single technique can offer; correlative microscopy has extraordinary potential for investigating materials properties, particularly their micromechanical characteristics [40].

**Fig. 2:**
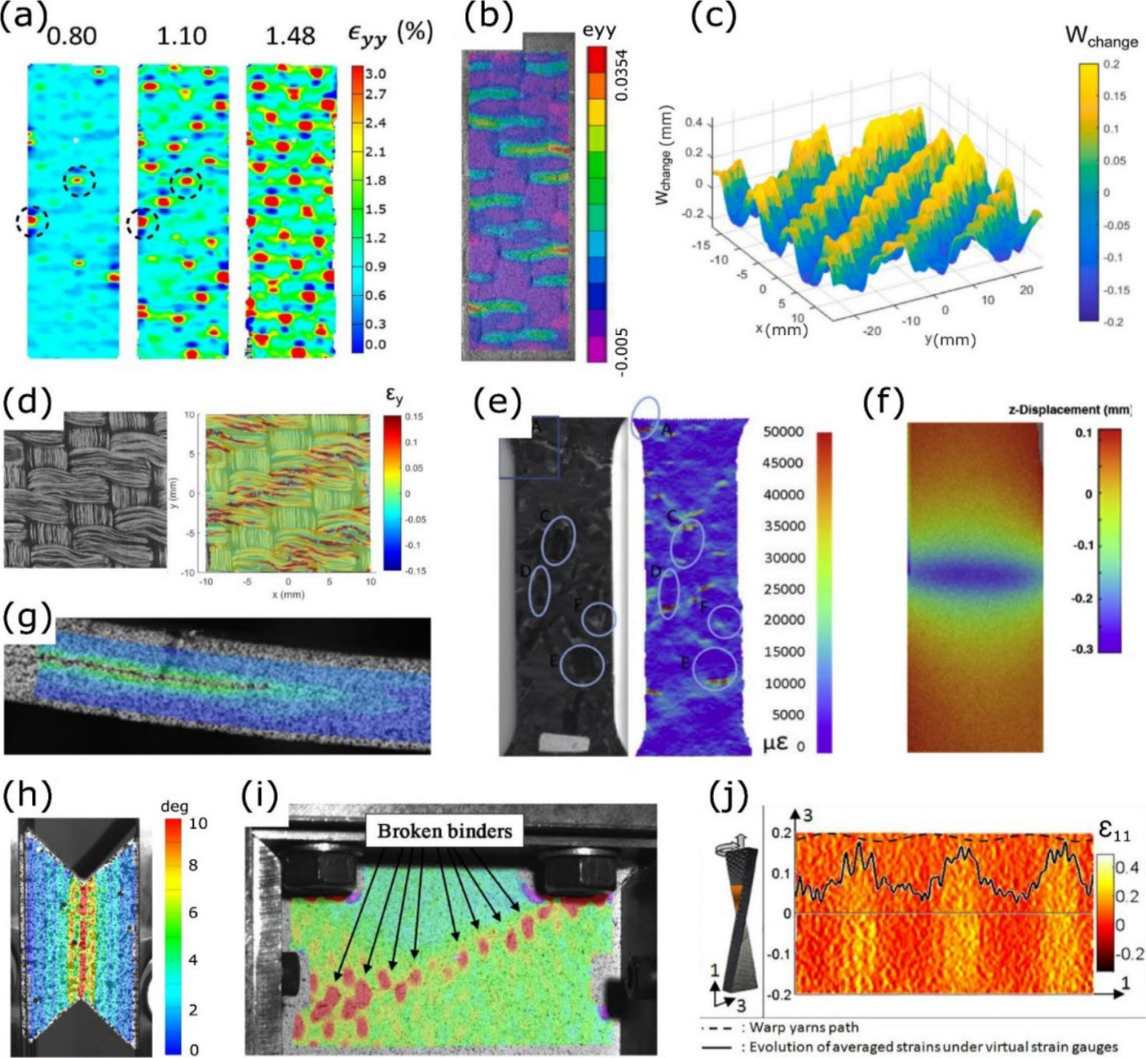
DIC applications for microscale and mesoscale structure, defects, and damage, **a** strain of z-binders due to failure in 3D woven polymer composite, **b** mesoscale strain concentrations linked to damage development for a 10 mm wide ceramic matrix composite at 900 °C, **c** topographical surface deformation of a twill woven composite, **d** varying local volume fraction within weave resulting in strain concentrations, **e** discontinuous fiber tows strain concentrations at the ends of fiber bundles, **f** delamination detection through out-of-plane motion of laminate composites, **g** crack front propagation observed with 2D-DIC shear strains during end-notch flexure test of carbon/epoxy woven laminate, **h** 30 mm long concentrated shear region influenced by architecture of 3D woven composite,** i** compression after impact for 3D woven composite showing broken z-binders as strain concentrations and (**j**) strain field for combined tension/torsion loading of 3D woven composite showing influence of the weave. Reproduced from [42] with permission from the publisher (Elsevier)

This review offers an oversight of in-situ optical and non-optical microscopies methods and techniques of taking images to incorporate with DIC, as well as software to improve images with microscopes for DIC to help readers to choose the most suitable corroborative techniques to address important fundamental/applied questions in polymer composite science and engineering with a view to high impact outcomes in technical and medical applications. In the near to medium term, we believe these will be combined with computational approaches to enhance product development in industry (Fig. 2) [41, 42].

## In-situ optical microscopy

### Polarized light optical microscopy

**Fig. 3:**
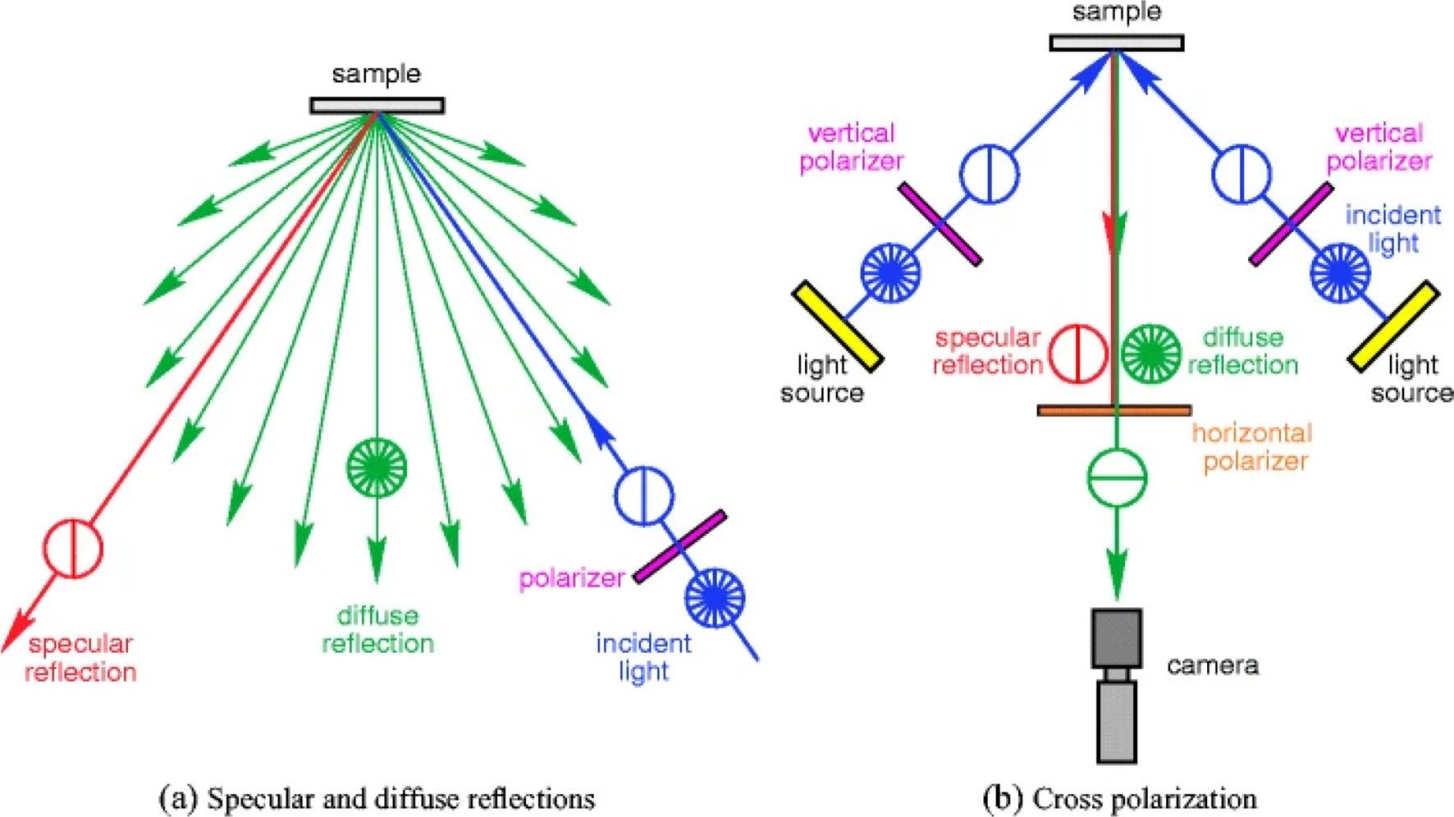
**a** Specular reflections maintain the polarization of incident light, while diffuse reflections do not. **b** The horizontal polarizer on the camera’s lens attenuates the vertically polarized specular reflections to avoid saturated pixels). Reproduced from [31] with permission from the publisher (Springer Nature)

Étienne-Louis Malus’s pioneering work on light polarization [43, 44], underpinned research and development of applications of polarized light in various scientific and technological fields (including spectroscopy, materials analysis, liquid crystal displays, and optical communication systems) [31, 45–47]. Polarized light is widely used for imaging thin sections of biological tissues (i.e., natural polymer composite materials), often in combination with polarization-sensitive fluorescence microscopy, polarization-sensitive hyperspectral imaging, polarization-sensitive multiphoton microscopy, etc. [48]. Polarized light optical microscopy (PLM) allows investigation of changes in microscale internal network structure of polymer-based materials caused by deformation [49, 50], furthermore, PLM is a valuable method for detecting and characterizing anisotropy in specimens that influence the polarization plane of light [51]. Cross polarization of light can enhance macroscopic, optical, and surface DIC measurements (Fig. 3) [31]; interesting studies have demonstrated enhanced image contract and mechanical testing using DIC in cellulose nanocrystal films [52] and bat wing skin [53].

### Stereo light microscopy

**Fig. 4:**
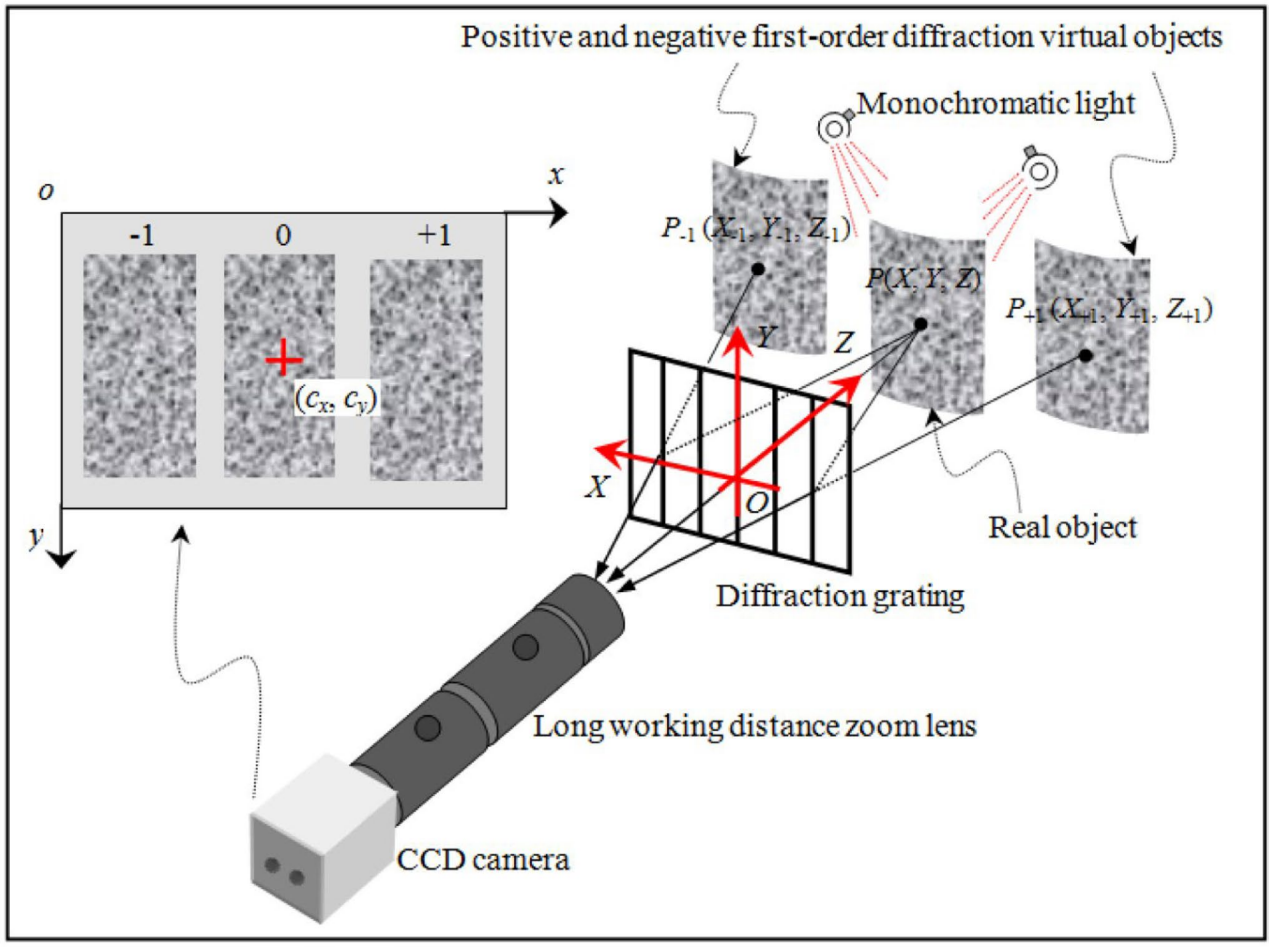
Schematic diagram of optical arrangement of the single-camera microscopic 3D-DIC method. Reproduced from [65] with permission from the publisher. © 2013 Optical Society of America

Stereo-DIC, also known as 3D-DIC [41], works over μm to m length scales with ns time resolution [54]. Stereo light microscopes (SLM) can be used to assist the DIC methods to measure deformation in small-scale sections [55], and stereo-DIC has been used to analyze various composite materials, including fiber-reinforced composites [56, 57], and given their potential in mechanics, materials research, and biological engineering, there is a strong demand for a low-cost, simple, and effective 3D-DIC technique for measuring small object shapes and deformations, which holds significant scientific value [58–64]. A diffraction assisted image correlation (DAIC) method (Fig. 4) can be used with samples ranging from submillimeter to a few centimeters and is much cheaper than existing systems (since both diffracted images are captured with one camera, synchronization issues are eliminated, and DAIC simplifies measurements by relying on diffraction rules for point correspondence, removing the need for intricate calibrations of the imaging system) [65]. Traditional binocular systems use parallax from two cameras for 3D spatial information but face challenges with precision on large objects, their processing algorithms are complicated by lens distortion, transformation, and calibration issues, making high-precision measurements difficult, and a new method utilizing a telecentric camera can circumvent these issues [66].

### Fluorescence microscopy and super-resolution fluorescence microscopy

**Fig. 5:**
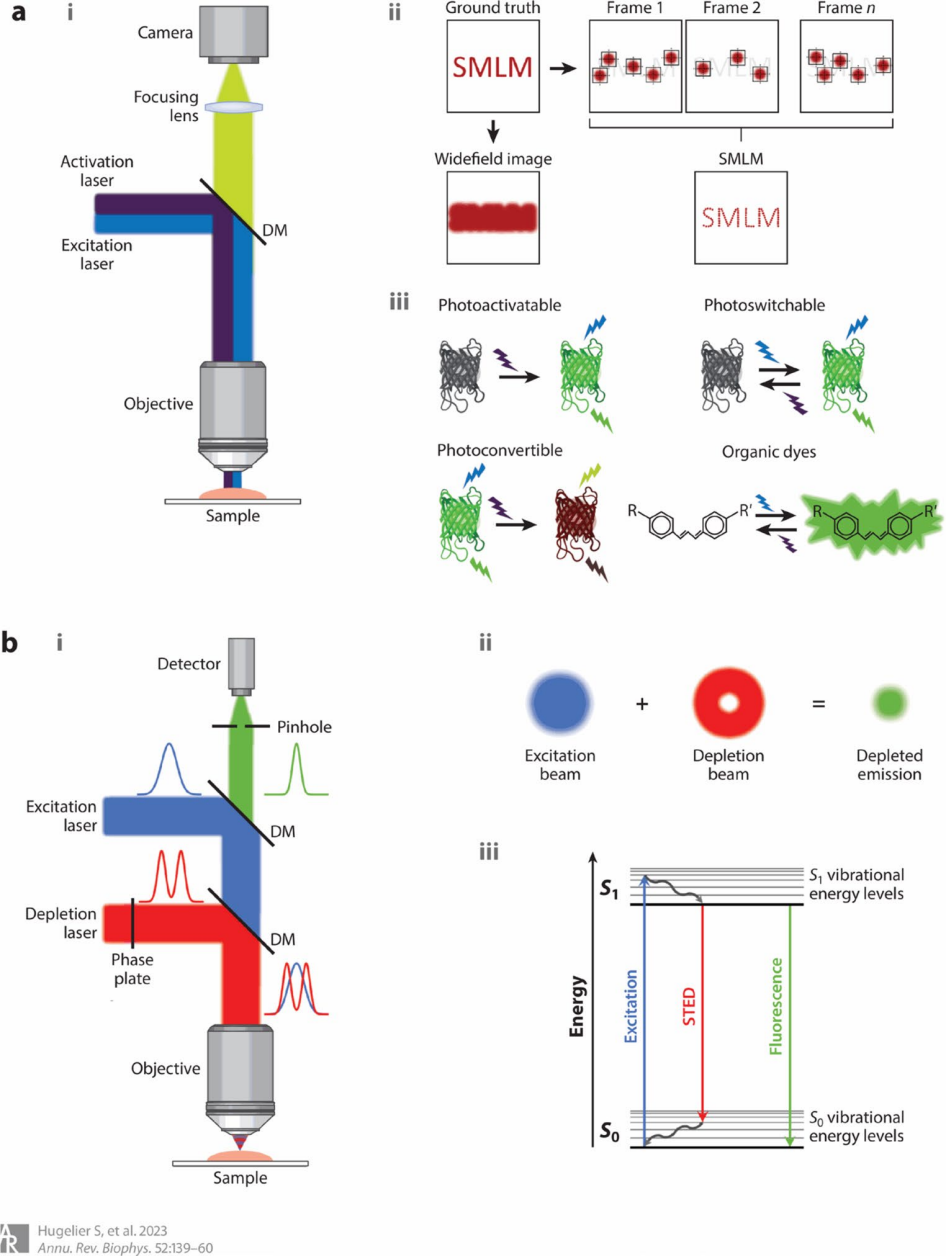
Schematic representation of the microscope modalities. **a** SMLM. The microscope setup (i), the working principle (ii), and some examples to achieve on/off switching of fluorescent proteins and organic dyes (iii) are shown. **b** STED microscopy. The microscope setup (i), the working principle (ii), and a Jablonski diagram of the STED excitation and emission (iii) are shown. Abbreviations: *DM* dichroic mirror, *SMLM* single-molecule localization microscopy, *STED* stimulated emission depletion. Reproduced from [85] with permission from the publisher (Annual Reviews)

**Fig. 6:**
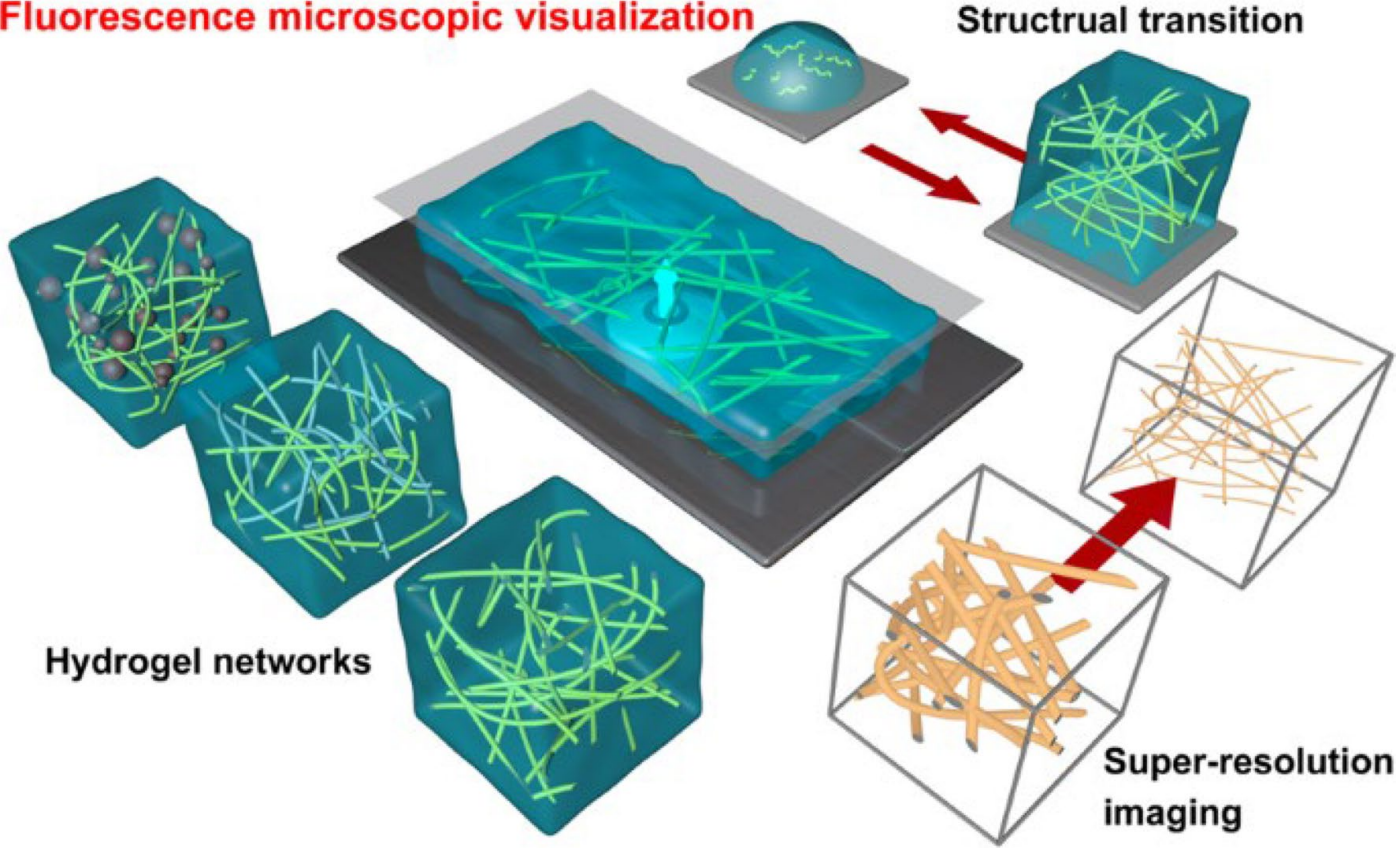
Schematic illustrating the visualization of different types of hydrogel networks, observation of structural transitions, and super-resolution imaging based on fluorescence microscopy. Reproduced from [73] with permission from the publisher (Springer Nature)

Fluorescence is a widespread phenomenon utilized, extensively worldwide for a variety of applications [67–69]. Fluorescence microscopy offers users high contrast, non/minimal-invasiveness, minimal preparation requirements, and ease of use [68]. In 2014, three chemists were jointly awarded the Nobel Prize for their contributions to advancing super-resolved fluorescence microscopy [70]. Originally aimed at biological systems by overcoming the diffraction limit of light, super-resolution fluorescence microscopy (SRFM) has now found significant applications in materials science, particularly in polymer research [71, 72]. This shift has prompted a growing number of studies focusing on developing and utilizing new fluorescence visualization techniques to deepen our insights into the specimens under investigation [73, 74]. These new methods include light sheet fluorescence microscopy (LSFM) [75, 76]; photoactivated localization microscopy (PALM) [77–79]; points accumulation for imaging in nanoscale topography (PAINT) [80, 81]; reversible saturable/switchable optical linear fluorescence transitions (RESOLFT) microscopy [82, 83]; single-molecule localization microscopy (SMLM) (Fig. 5) [84–86]; stimulated emission depletion (STED) microscopy [72, 87, 88]; stochastic optical fluctuation imaging (SOFI) [89]; stochastic optical reconstruction microscopy (STORM) and direct STORM (dSTORM) reconstruct the positions of all fluorophores employed to achieve images with a resolution of 20 nm which makes them valuable tools for biological imaging applications [90, 91]; and structured illumination microscopy methods [92]. We have now entered an era where developments in fluorescence imaging strategies (e.g., staining techniques like post-staining following force loading, pre-treatment, and activation of turn-on fluorescence) [79], and quantification methods [93, 94] offer opportunities for super-resolution fluorescence microscopy (SRFM) to investigate the mechanical properties of polymer composite-based materials and deliver exciting results (Fig. 6) [73, 74].

### Confocal laser scanning microscopy (CLSM)

**Fig. 7:**
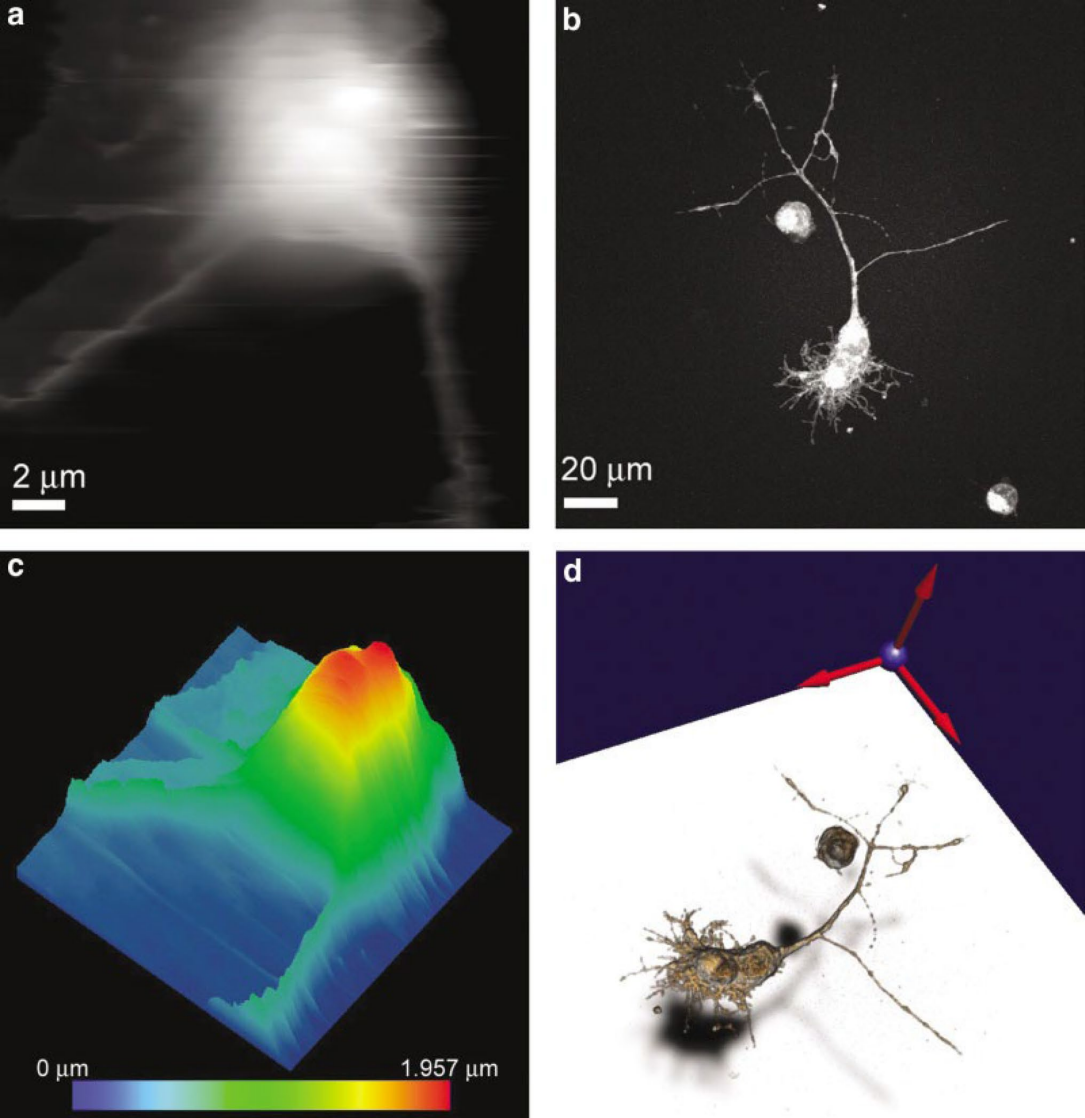
Examples of visualization of AFM and CLSM data. **a**: AFM range image (gray scale proportional to the elevation); **b**: CLSM maximum intensity projection image; **c**: AFM pseudo-colored isometric view; **d**: CLSM volumetric imaging. The AFM images show the cell body of a live neuronal cell, while CLSM images show the whole live neuron stained with FM 1–43. Reproduced from [101] with permission from the publisher (John Wiley & Sons, Inc.)

High quality confocal microscopy [95] underpins the mechanical scanning confocal laser microscope [96]. Confocal laser scanning microscopy (CLSM) is applicable to polymer-based materials [97–99], and is utilized by biologists to delineate biological pathways, comprehend intracellular mechanisms, and observe the general structures of living cells [100, 101]; it is capable of high-resolution functional cell imaging, albeit restricted to a depth of about 300 μm [102]. AFM typically offers sub 1 nm resolution [103–106], hence, combining AFM and CLSM is interesting for biological applications [100, 101, 107], collecting much more precise 3D images and in-depth analysis of a specimen’s structure (Fig. 7) [100–102, 107–110]. One novel technique for improving 3D image capture by LSCM involves combining it with DIC, referred to as the confocal-DIC method [110]; while it is predominantly employed to examine biological samples and colloidal particles [111], it can also aid in characterizing porous structures and nanostructures [112]. Some microscopy-DIC methods are listed in Table 1.

**Table 1:** Examples of optical microscopy-DIC methods

Methods	Size/pixels	Techniques	Software	References
Polarized light microscopy + SEM	1–200 µm	Step-and-shoot	Fiji	[49]
Polarized light microscopy + SEM	≈150 µm	PLM/INT	NIS Elements	[50]
Polarized light microscopy	0.73 μm	Cross-polarized		[51]
Stereo light microscopy	2456 × 2058 pixels	Scheimpflug cameras for 3D microscopic DIC	Ncorr	[54]
Stereo light microscope	0.063 × 0.063 mm	2D DIC (MDIC)	Elite Software	[113]
Light sheet fluorescence microscopy (LSFM)	532/580 nm	OptoRheo	ImageJ/Fiji	[76]
Confocal microscopy	0.5 μm	Hand-drawn contouring system	SURPASS, Leica LAS X 3D, IMARIS	[114]

## In-situ non-optical microscopy

### SEM

**Fig. 8:**
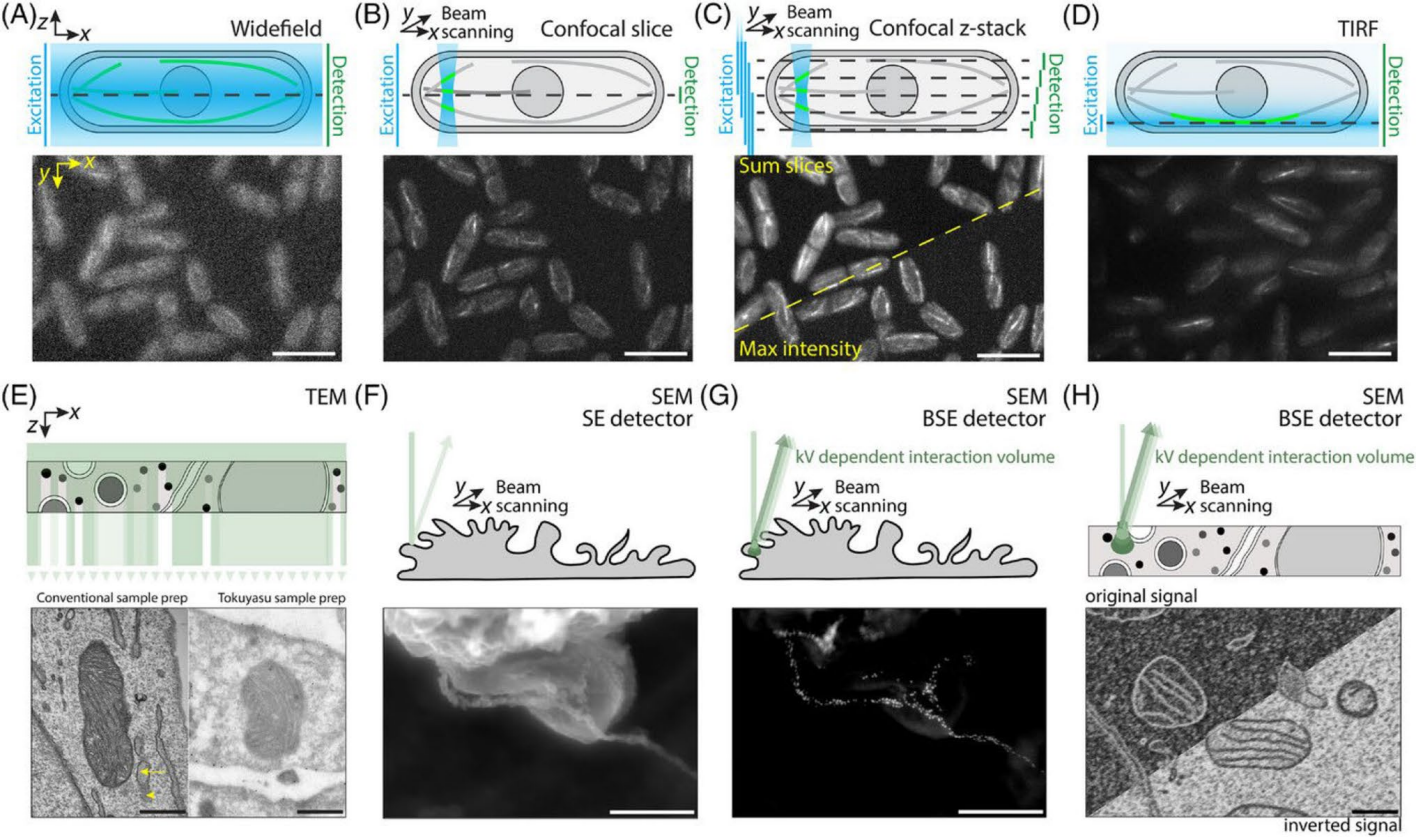
Image formation in fluorescence and electron microscopy. **A** Widefield microscopy captures live *Schizosaccharomyces pombe* cells expressing sfGFP-tubulin, illuminating the entire sample volume simultaneously. Out-of-focus fluorescence can obscure fine details. **B** Confocal microscopy scans individual diffraction-limited laser spots (laser-scanning) or sweeps them (spinning disk) to avoid out-of-focus light, enhancing contrast and detail across sample depths. **C** Confocal slices can be compiled into a 3D ‘z-stack’, which can be projected as a single image through summed or maximum intensity values. **D** TIRF microscopy employs an evanescent field that illuminates only a few hundred nanometers from the coverslip, capturing fluorescence primarily from structures near the coverslip, differing from (**A**)–(**C**). All fluorescence scale bars = 10 μm. **E** TEM reveals mitochondria in a thin embedded section, comparing different preparation protocols: conventional (electron dense) and Tokuyasu (electron lucent). Tokuyasu image by I. J. White. **F** SEM detects exocytosis events on endothelial cells, visualizing Von Willebrand factor strings. **G** SEM with backscattered electron detection highlights gold-labelled antibodies on Von Willebrand factor. Images by K. O'Neill and D. Cutler. **H** SEM with BSE of a resin-embedded thin section shows heavy metal areas producing stronger signals (light) compared to lighter regions. This method often inverts data for clearer comparison, with green ellipses indicating interaction volumes of the electron beam at varying voltages. All EM scale bars = 500 nm. Reproduced reference [93] with permission from the publisher (John Wiley & Sons)

SEM images samples over length scales ranging from nm to cm [115], typically via secondary electron imaging (SEI) and backscattered electron imaging (BEI) (Fig. 8) [93]. The application of DIC-SEM is attributed to the synergistic benefits of combining these two powerful techniques, as well as the continuous advancements in the underlying technologies and their increasing accessibility to researchers across various fields [34, 116, 117]. DIC-SEM has been used to study topics such as grain boundary sliding, deformation twinning in materials, crack propagation phase transformations, as well as characterizing the mechanical properties of individual grains, inclusions, and other microstructural features within a material [16, 34, 118–121].

### Transmission electron microscopy (TEM) and scanning transmission electron microscopy (STEM)

**Fig. 9:**
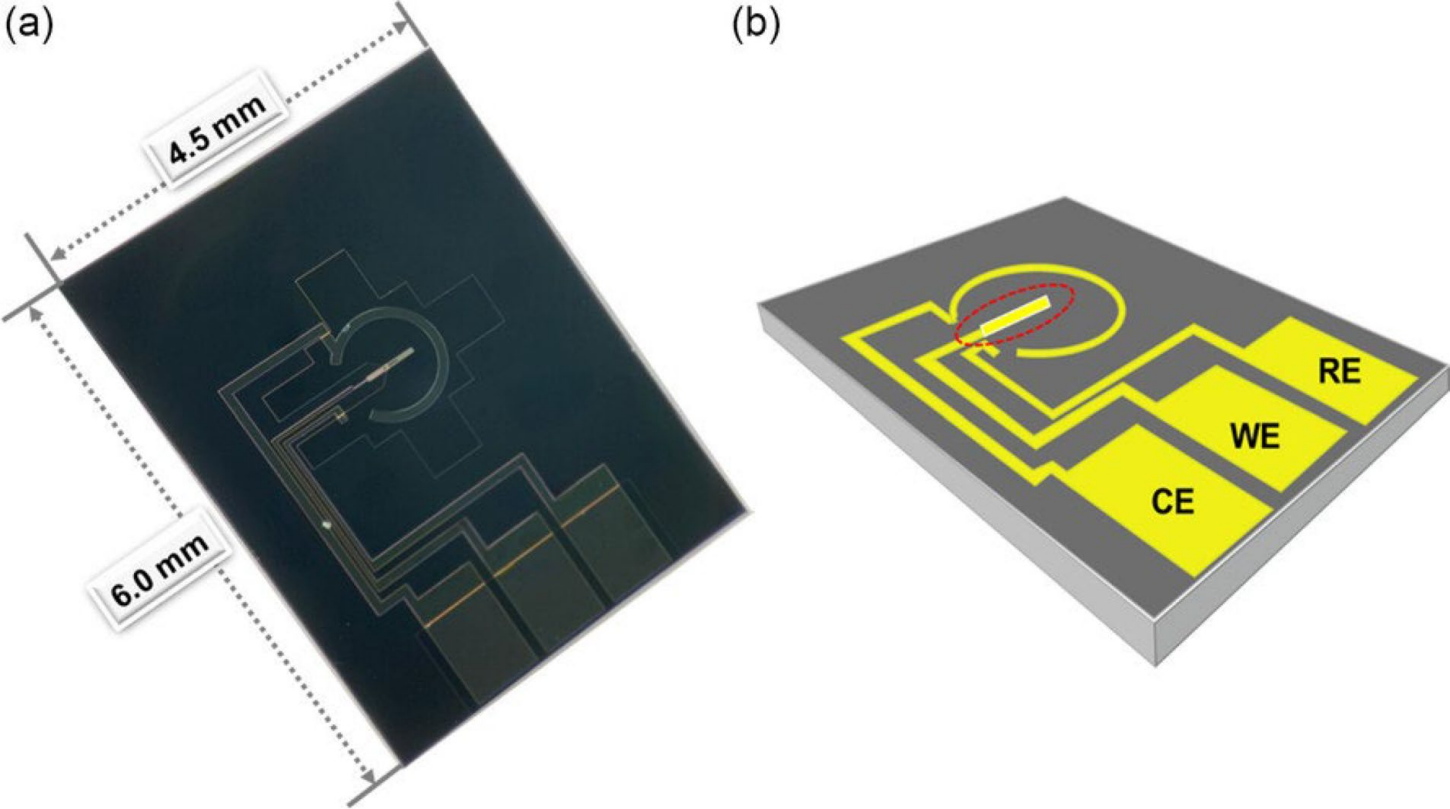
Picture of the in situ electrochemical TEM chip. **a** Schematic view of the in situ electrochemical TEM chip, and **b** the viewing window was indicated by the red dashed circle. Reproduced from [136] with permission from the publisher (John Wiley and Sons)

**Fig. 10:**
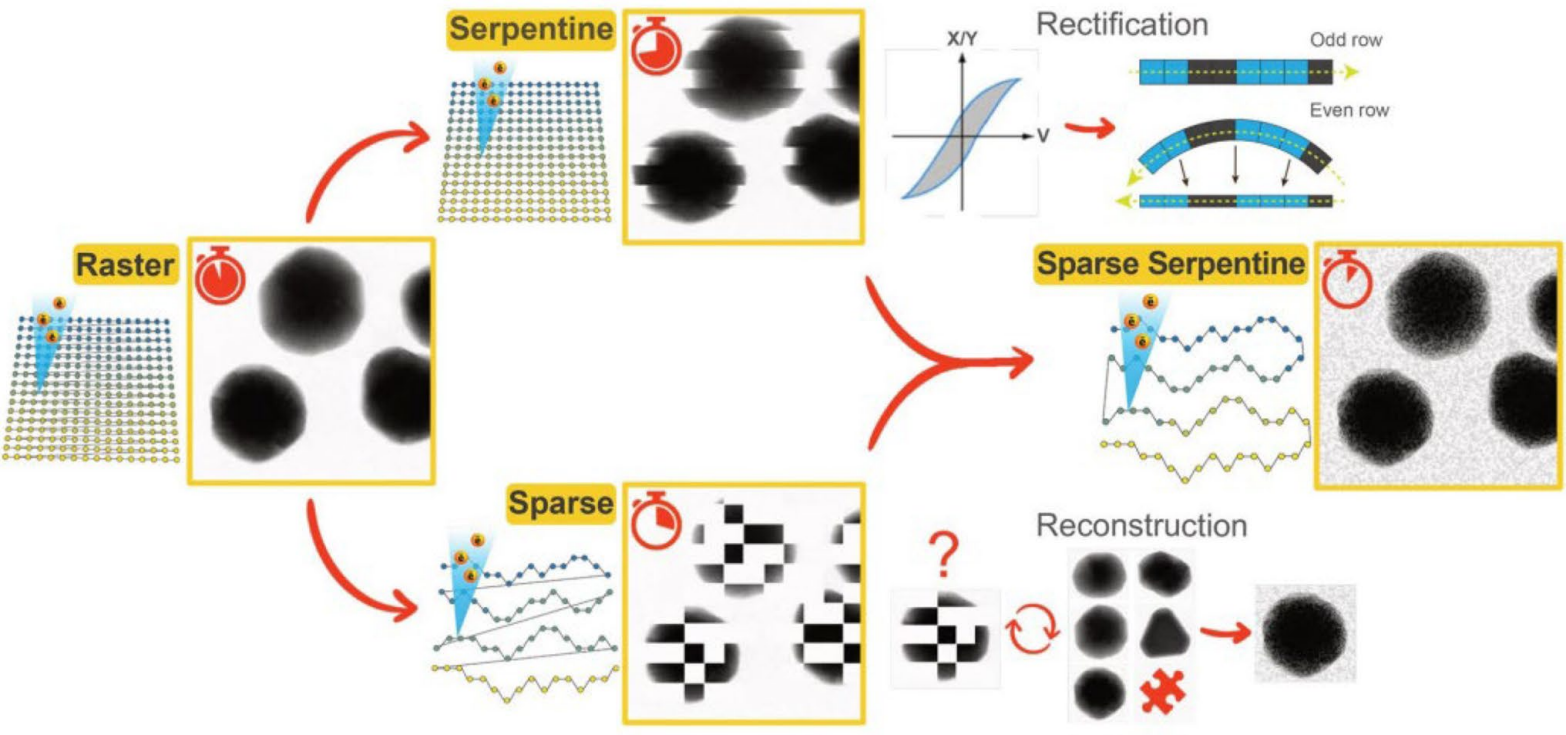
Schematic illustration of different scanning strategies and their relative acquisition time. The standard pixel-by-pixel raster scan of Scanning Transmission Electron Microscopy (STEM) includes the addition of a 'flyback' time to relocate the beam at the beginning of the next row. Via a serpentine scan, the frame rate can be improved by avoiding any dead time τ, but the rectification of odd and even rows is required to compensate for hysteresis effects of the magnetic scan coils. Sparse imaging results in the recording of fewer pixels through a random-walk scan thus reduce the total time. Here, a reconstruction algorithm is needed to “inpaint” the full frame. Both approaches can be combined to achieve the highest possible STEM image acquisition speed while avoiding an increase in electron dose. Reproduced from [134] with permission from the publisher (Springer Nature)

TEM is extensively utilized for analyzing and imaging nanoscale samples [31, 115, 122–124]; high-resolution TEM (HRTEM) has a resolution of ≈ 0.5 Å (0.050 nm); cryo-TEM rapidly freezes samples for analysis without inducing changes like agglomeration or deformation. In situ TEM demands special materials—electron-transparent and ultrathin samples—limiting the range of materials tested, with imaging capped at ≈30 frames/second. These techniques necessitate controlled conditions, such as a vacuum SEM/TEM environment, as opposed to reactive air, and recent studies have incorporated DIC measurements in TEM (Fig. 9) [124–126]. STEM merges SEM and TEM modes, has atomic level resolution [127], and has been used to analyze phase separation in polymer blends [128] and a variety of other materials (Fig. 10) [129–135].

### X-ray microscopy

**Fig. 11:**
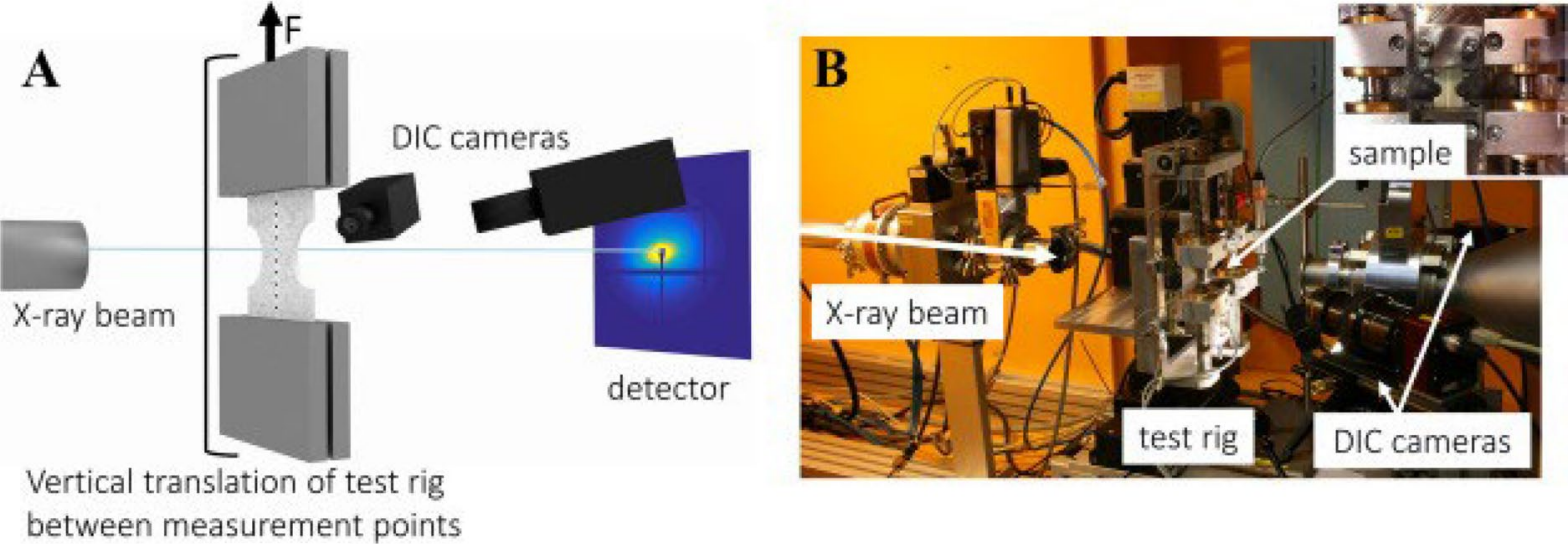
Experimental setup. **A** Schematic illustration of a sample tested in tension and simultaneously monitored with DIC and SAXS or WAXS at 10 discrete vertical positions in the horizontal-centre of the sample. The sample is subjected to continuous tensile loading in the test rig and the whole rig is translated vertically to move the sample between the measurement points inside the beam. **B** Photograph of the experimental setup at the I911-4 beamline (MAX IV Laboratory, Lund University, Lund, Sweden). Reproduced from [138] with permission from the publisher (Elsevier BV)

**Fig. 12:**
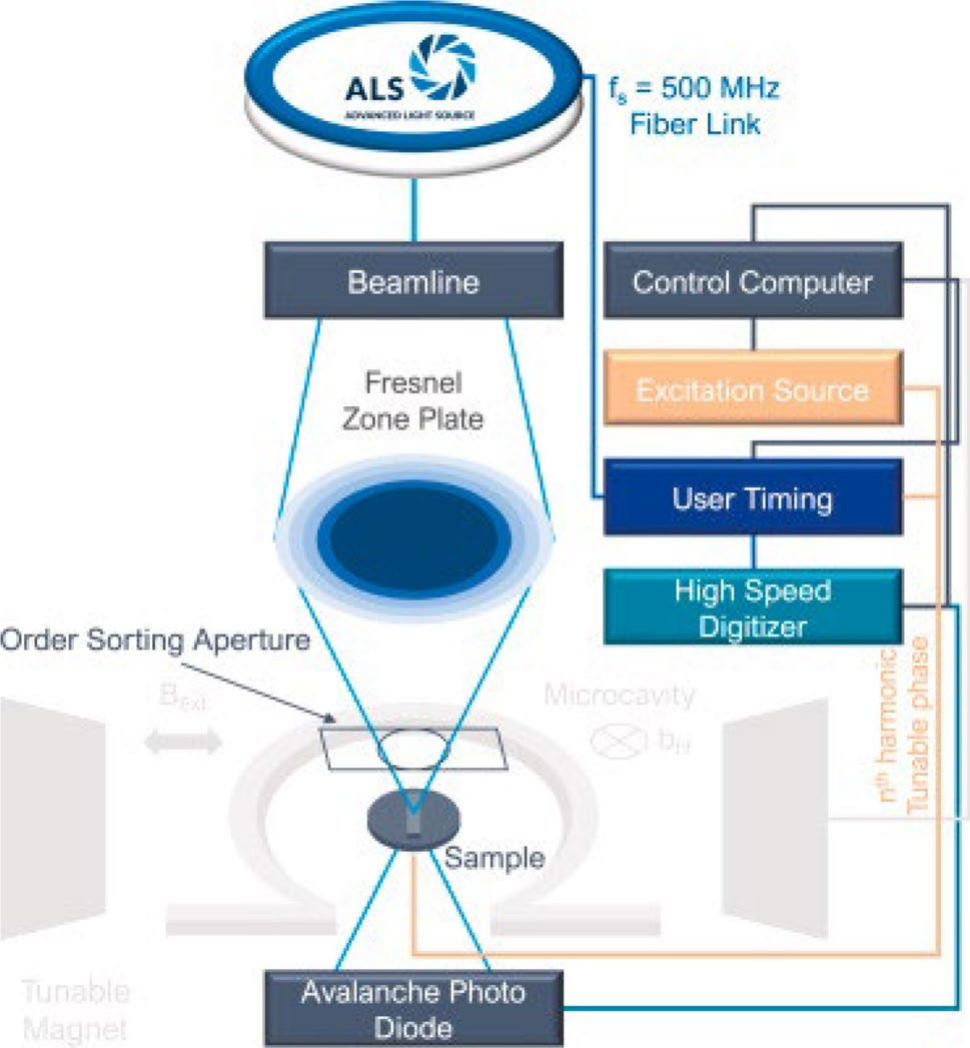
Schematic representation of the TR-STXM setup realized at ALS beamline 11.0.2.2. The STXM is situated in a high-vacuum chamber. Gray colored parts are optional components for (dynamic) magnetic measurements. Reproduced from [142] with permission from the publisher (Elsevier BV)

The intricate morphology of many modern materials spans various length scales, requiring multi-scale modeling to understand mechanical behavior from atomic to macroscopic levels [137]. Synchrotron X-ray facilities have improved our ability to study material structures at various length scales using small and wide-angle X-ray scattering (SAXS and WAXS); indeed, in situ mechanical testing in combination with SAXS/WAXS enables the measurement of nanoscale deformations that can be combined with DIC to analyze deformation across multiple length scales simultaneously (Fig. 11) [138–140]. X-ray imaging has transformed microscopy, and in situ soft X-ray Scanning Transmission X-ray Microscopy (STXM) has a spatial resolution of ≈25 nm [126, 141–143] which is applicable to biological samples (Fig. 12); cross-compatibility with CLSM/TEM enables mapping of macromolecule structure/composition through multi-microscopy approaches, which can image complex systems, e.g., biofilms [144]. While advanced microscopy offers many benefits, it can be time-consuming and involve complex digital image algorithms [145]; new software like STXM_deconv helps users with limited image processing skills [146], and Gaussian mixture (GM) and Bayesian Gaussian mixture (BGM) clustering methods operate based on similarity and proximity rather than traditional algorithms [147]. Employing these strategies demonstrates X-ray microscopy’s ability to enable mapping and analyzing elemental structures, aiding DIC method in investigating the chemical/micromechanical properties of polymer-based materials.

### Atomic force microscopy (AFM)

**Fig. 13:**
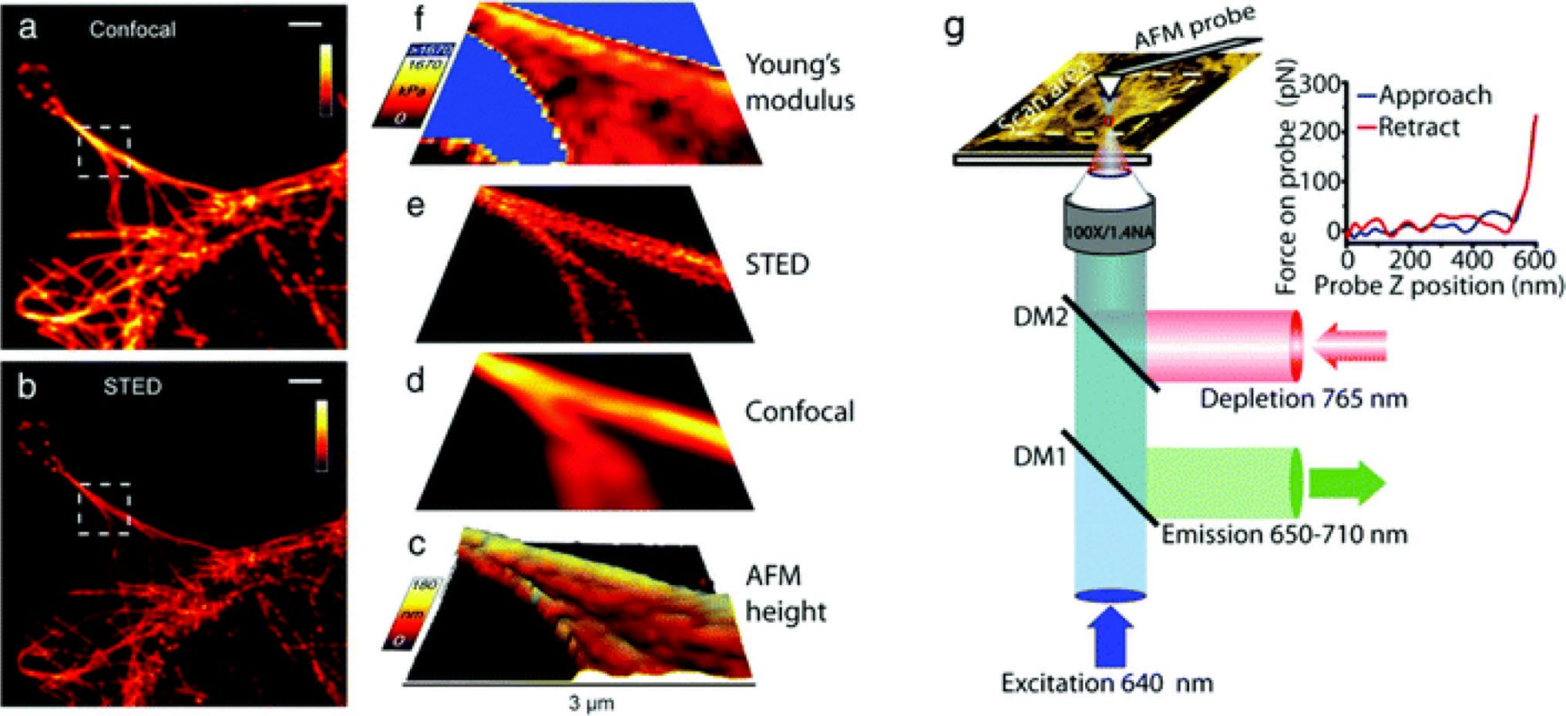
**a**–**f** Correlative AFM + STED imaging of Cos7 cells labelled with Atto 647 N. **a** Confocal raw image, **b** STED raw image, **c** 3D rendered view of AFM measured height extracted from AFM force curves, deconvolved (**d**) confocal and (**e**) STED images, and (**f**) an elasticity map calculated from AFM force curves. **g** Schematic diagram of a combined AFM/STED imaging set-up. The AFM cantilever is aligned such that STED and AFM have a common scan area. Fluorescence excitation pulses are combined with depletion pulses using a dichroic mirror (DM2), and fluorescent emission is separated using a dichroic mirror (DM1). AFM images are acquired by translating the sample. For each pixel a force curve is measured by approaching the tip toward the sample and recording the tip–sample interaction force as a function of the cantilever z-position (see inset), and the Young’s modulus is estimated from the gradient. Scale bars in (**a** and **b**): 2 μm. (**a**–**f**). Reproduced from [150] with permission from the publisher (Royal Society of Chemistry)

Atomic Force Microscopy (AFM) has nm scale resolution via a surface probe technique that enables topographical and nanomechanical measurements (potentially under physiological conditions) [103, 148] and recent advances enabled studies of multiparametric heterogeneity of materials [149, 150]. Combination with Total Internal Reflection Fluorescence Microscopy (TIRFM) [151] and Scanning Near-Field Optical Microscopy (SNOM/NSOM) [152] has enabled interesting biological applications, e.g., (Fig. 13). It has also been possible to demonstrate simultaneous imaging and nanomanipulation [149, 150]. Some non-optical microscopes-DIC methods are listed in Table 2.

**Table 2:** Non-optical microscopy-DIC methods

Methods	Size/pixels	Detector	Software	Techniques	References
SEM	25 × 25 pixels		ARAMIS	BEI, SEI	[34]
SEM		Air brush	GOM Correlate	SENT	[118]
SEM	Micro scale	Electron backscattering diffraction (EBSD)	GOM Correlate	HKL channel 5	[119]
SEM	micro-scale	A backscattered electron (BSE)	Avizo v2020	Operando	[153]
Focused ion beam (FIB)	10–15 nm			Liquid gallium (Ga)	[154]
SEM	≈1 μm	Back Scattered (BSD), Secondary Electron (SED)	VIC-2D software	Step-and-shoot	[120, 155]
SEM	≈10 nm	Secondary electron emission, back scattered electron emission	Wolfram Mathematica §R 10	Stereo pair	[121]
SEM	≈10 nm	MD (mirror detector) for BSE imaging	CASINO V2.51	Very low voltage (VLV)	[156]
STEM + X-ray ptychography	128 × 128 pixels	(EMPAD)		Annular dark field (ADF)	[157]
SEM		SE, BSE, EBSD, EDS	VEDDAC		[158]
TEM	≈2 nm	Laplacian of Gaussian (LoG)	VIC-2D	Particle Tracking (PT)	[124]
STEM	512 × 512 pixels	High angle annular dark field (HAADF)	Nuxutra Image-Inpainting	Nuxutra Image-Inpainting, Fiji ImageJ, Digital Micrograph	[134]
STEM	(≈0–40 mrad)	Annular dark field (ADF)	STEMsim software package	High angle scattering of electrons captured	[135]
STXM	25 nm			Operando	[126]
STXM (three types)	< 1 mm			Talbot-carpet	[143]
DIC-SAXS/WAXS	300 × 300 μm^2^	PILATUS 2 M	ImageJ v1.50i/VIC-3D7	plugin OrientationJ	[138]

## Software

**Fig. 14:**
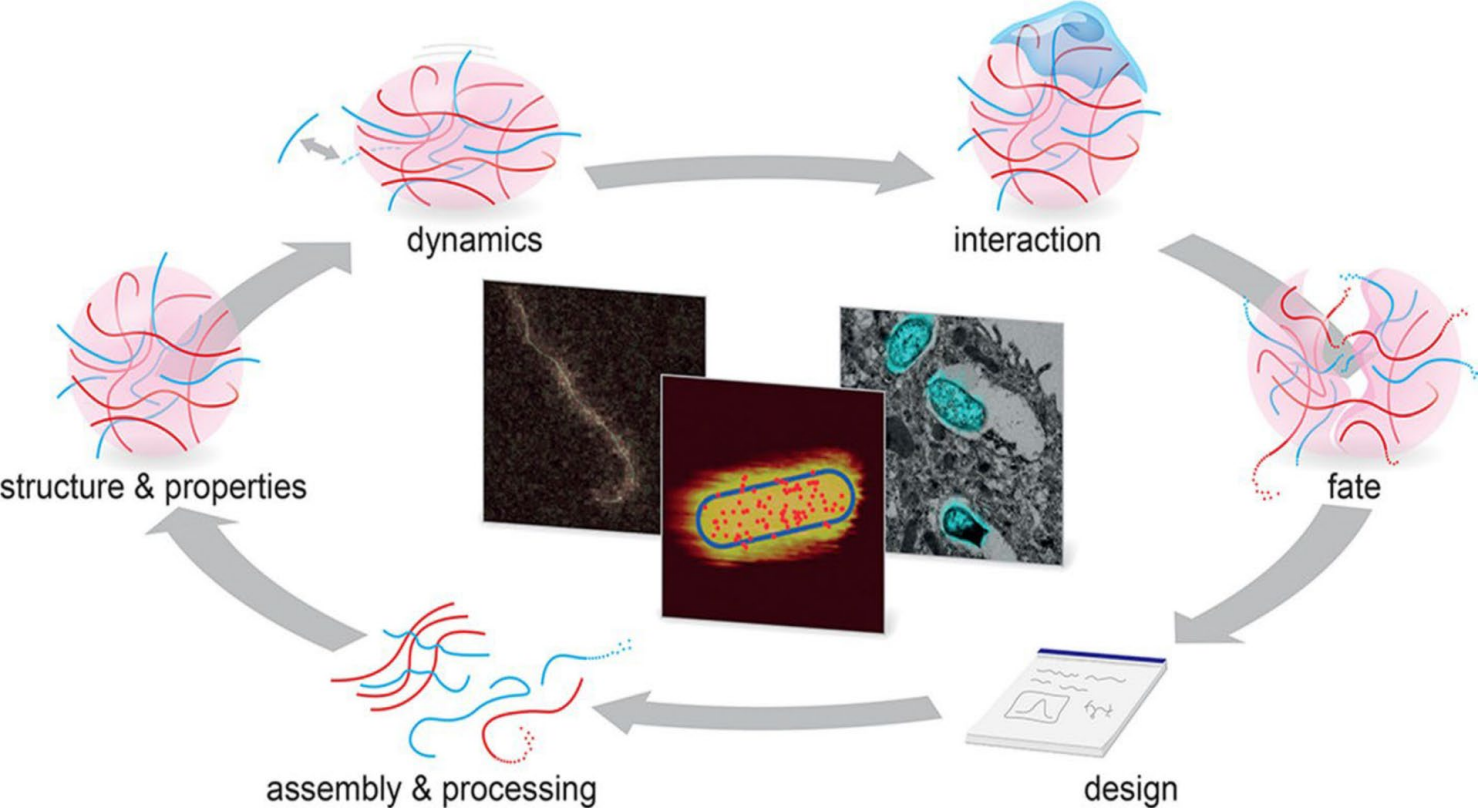
Steps in the life cycle of polymeric materials that correlative microscopy can contribute to study and understand. Reproduced from [40] with permission from the publisher (John Wiley & Sons)

The DIC methodology employed for measuring mechanical properties involves image acquisition, processing, and correlation; free and commercial software have been created for analysis of DIC data, examples of which are listed in Table 3 [52, 113, 159–164]. These resources are versatile and can be utilized across various research fields where understanding structure–property relationships are of critical importance, and we foresee them playing an increasingly important role in research and development in academia and industry (Fig. 14).

**Table 3:** Examples of DIC Software

Software	Commercial/open source	Implement	Institute/company	Application	References
ARAMIS	Commercial	Python		Uniform illumination of the specimen surface	[113, 159]
CorreliSTC	Commercial		Airbus Group Innovations	2D, 3D	[159]
Dantec Dynamics	Commercial	MATLAB		2D, 3D	[163]
Eikosim	Commercial	MATLAB		2D, 3D	[163]
Elite		Python		2D, 3D	[113]
GOM	Commercial	C + + , MATLAB		3D	[159–163]
ISI-Sys VIC	Commercial	C + + , MATLAB		2D, 3D	[160]
LaVision StrainMaster	Commercial	MATLAB	Max Planck Institute and Laser Laboratory in Gottingen	2D, 3D	[159, 161–163]
MatchID-2D	Commercial	MATLAB		2D, 3D	[159, 162, 163]
Q-400	Commercial		Dantec Dynamics	3D	[159]
TEMA	Commercial			2D, 3D	[159]
VEDDAC	Commercial			3D	[158]
Vic-2D	Commercial		Correlated Solutions Inc, USA	2D displacements	[120, 155] [159, 161]
Video Gauge™	Commercial		Imetrum	High stress, crack opening or other discontinuities	[159]
ALDIC	Open source	MATLAB		2D	[161–163]
ADIC2D	Open source	MATLAB		2D	[162, 163]
ADIC3D	Open source	MATLAB		3D	[163]
Avizo v2020	Open source	MATLAB		3D	[153]
DIC Engine (DICe)	Open source	C + +	Sandia National Laboratories, Albuquerque, New Mexico	GUI 2D, 3D	[159–163]
Digital Micrograph	Open source	MATLAB	Gatan Ametek, CA, USA- University of Wollongong, NSW, Australia	2D	[134]
dolfin_dic	Open source	Python	École Polytechnique, Palaiseau, France	2D, 3D	[159]
iCorrVision-2D	Open source	Python		2D, material characterization, perform J -Integral, kinematic parameters, investigate the deformation homogeneity of mechanical samples	[162]
iCorrVision-3D	Open source	Python		3D	[163]
ImageJ	Open source	MATLAB	Wayne Rasband, National Institute of Health, Bethesda, MD, USA	2D, 3D	[76, 153, 165]
MultiDIC	Open source	MATLAB		3D	[163]
NCorr	Open source	MATLAB C + +	Georgia Institute of Technology, Atlanta, Georgia	Graphical User Interface (GUI), 2D, behavior of the foam under compression	[54, 153, 159–163, 166]
NIS Elements 4.0			Laboratory imaging, Czech Republic		[50]
PReDIC	Open source	MATLAB		2D	[163]
pydic	Open source	Python	University of Limoges, Limoges, France		[159, 161]
py2DIC	Open source	Python	(University of Rome La Sapienza, Rome, Italy	GUI 2D	[159, 161–163]
pyxel	Open source	Python	Institut National des Sciences Appliquées de Toulouse, Toulouse, France	Mechanics, 2D	[159]
RealPi2dDIC	Open source	Python		2D	[161–163]
µDIC	Open source	Python		2D	[160–163]
Ufreckles	Open source	MATLAB		2D	[161, 163]
YaDICs	Open source	C + +	Laboratoire de Mécanique de Lille, Lille, France	Kinematics, 2D, 3D	[159, 162, 163]
YADICS	Open source	MATLAB		2D	[161–163]

## Combination with other techniques

The exciting properties of natural/synthetic composites highlights the importance of understanding their mechanical behavior [167]. DIC can analyze the behavior of such composites under different loads and moreover, be combined with other mechanical methods for greater insights [168], where DIC provides high-resolution images and insights about the micromechanical characteristics of such materials [169]. The combination of DIC and finite element analysis (FEA) has been used to investigate dentin micromechanics [170] or those of human soft tissues [171], to conducting fatigue tests on composites [172], to study distributed fiber optic sensors (DFOS) in concrete structures [173], or indeed small punch tests (SPT) for specimen mapping [174]. Nanoindentation measurements are well established in the analysis of polymer-based materials and their composites [175–178]. A nanoindentation method allows for local mechanical characterization of materials at micro and nanometer scales; this technique has been applied across diverse fields, including biology, engineering, geology and materials science [167, 179]; however, despite its widespread use, this method cannot independently account for the elastic, hardness [179, 180], and chemical [181–183] properties of shale due to the inherent complexity of the source rocks, but the combination with DIC can effectively resolve these issues. Some 3D imaging technologies (e.g., computed tomography (CT) [184–187], etc.) combined with DIC algorithms offer opportunities assess internal displacement/deformation information of materials-digital volume correlation (DVC), this is becoming increasingly important in the field of analyzing material microstructures, and we point the interested reader towards excellent review articles [188–191]. Some examples of DIC combined other techniques are listed in Table 4.

**Table 4:** Examples of application of DIC combined with other techniques to analyse composites

Combination methods	DIC measure	Combined-method measure	Microscopy	Specimen	References
DIC-FEA	Strain	Stress	Optical	Dentin	[170]
DIC-fatigue test	Map strain	Stiffness/absorb energy		E-glass fiber	[172]
DIC-DFOS	Strain	Crack monitoring	Optical	Concrete structures	[173]
DIC-SPT	Elastic and plastic	Tensile	Optical	Steel	[174]
DIC-Nanoindentation	Elastic/hardness		SEM	Rocks	[179, 180]
DIC-Nanoindentation	Stiffness	Viscoelasticity	AFM	Composite	[167]

## Conclusion

The mechanical properties of polymer-based materials is one of the factors that make them ubiquitous in our everyday lives [3, 4]. DIC can be integrated with both traditional and innovative techniques to validate and enhance mechanical testing studies, providing deeper insights into the material’s characteristics and factors influencing them. While effective in controlled lab settings, DIC studies undertaken in natural environments are challenging owing to a variety of complications including uneven illumination, shadows, blurring, and noise, which can hinder its effectiveness and may be addressed in future research. In this review we highlight the viability of both optical and non-optical microscopic methods for obtaining high-quality images using DIC across various length and time scales which have attracted significant attention from researchers in academia and industry. We believe such techniques will play an important role in the future of materials science and engineering [192]; indeed DIC is one of a variety of computational approaches that can be applied to generate large datasets to feed into models that enable the development and production of advanced materials to precisely designed properties potentially employing AI/ML approaches to facilitate this [193, 194].

## Data Availability

Not applicable.
